# A predictive model for personalization of nanotechnology-based phototherapy in cancer treatment

**DOI:** 10.3389/fonc.2022.1037419

**Published:** 2023-01-04

**Authors:** Eli Varon, Gaddi Blumrosen, Orit Shefi

**Affiliations:** ^1^ Faculty of Engineering, Bar-Ilan University, Ramat Gan, Israel; ^2^ Bar-Ilan Institute of Nanotechnology and Advanced Materials, Bar-Ilan University, Ramat Gan, Israel; ^3^ Department of Digital Medical Technologies, Holon Institute of Technology, Holon, Israel; ^4^ Department of Computer Science, Holon Institute of Technology, Holon, Israel; ^5^ Gonda Multidisciplinary Brain Research Center, Bar-Ilan University, Ramat Gan, Israel

**Keywords:** cancer, radiation, prediction medicine, biomedical model development, PDT (photodynamic therapy), PTT (photothermal therapy), personalized medicine

## Abstract

A major challenge in radiation oncology is the prediction and optimization of clinical responses in a personalized manner. Recently, nanotechnology-based cancer treatments are being combined with photodynamic therapy (PDT) and photothermal therapy (PTT). Predictive models based on machine learning techniques can be used to optimize the clinical setup configuration, including such parameters as laser radiation intensity, treatment duration, and nanoparticle features. In this article we demonstrate a methodology that can be used to identify the optimal treatment parameters for PDT and PTT by collecting data from *in vitro* cytotoxicity assay of PDT/PTT-induced cell death using a single nanocomplex. We construct three machine learning prediction models, employing regression, interpolation, and low- degree analytical function fitting, to predict the laser radiation intensity and duration settings that maximize the treatment efficiency. To examine the accuracy of these prediction models, we construct a dedicated dataset for PDT, PTT, and a combined treatment; this dataset is based on cell death measurements after light radiation treatment and is divided into training and test sets. The preliminary results show that the performance of all three models is sufficient, with death rate errors of 0.09, 0.15, and 0.12 for the regression, interpolation, and analytical function fitting approaches, respectively. Nevertheless, due to its simple form, the analytical function method has an advantage in clinical application and can be used for further analysis of the sensitivity of performance to the treatment parameters. Overall, the results of this study form a baseline for a future personalized prediction model based on machine learning in the domain of combined nanotechnology- and phototherapy-based cancer treatment.

## 1 Introduction

Cancer remains a leading cause of death globally (1). The conventional methods of treatment offered are radiation (2), chemotherapy (3), immunotherapy (4), surgery, and recently nanotechnology (nanomedicine and nano-processes) (5). Every cancer treatment can be defined and evaluated based on its efficiency, selectivity, side effects, and economic cost (6). However, combining predictive models and advanced machine learning methods with these cancer therapies may enhance their overall efficiency and selectivity, as well as the safety of the patient.

Radiation Therapy (RT), also known as radiotherapy, is a non-surgical intervention frequently used in cancer treatment (2). This method is based on a high-level focused dose of radiation directed toward the tumor. This deposit of high-energy radiation kills cancer cells or decelerates their growth by damaging their DNA (2). Nevertheless, the challenges of RT include damage to tumor- proximate normal cells, the inability to radiate minor tumors out of scope of the imaging scans, patient movement, and low oxygen supply (7, 8). Therefore, many researchers are working on the development of targeted radiation methods to deliver a higher dose of radiation to the tumor with improved selectivity.

Recently, the combination of nanotechnology with laser radiation has been demonstrated to represent a safe set of modalities for tumor destruction with high specificity (9). In particular, this involves the use of light-controlled nanoparticles (NPs) that can be activated *via* a light of a specific wavelength to form highly efficient and selective systems in the nanometer range (10). These NPs accumulate specifically within tumors due to the Enhanced Permeability and Retention (EPR) effect (11). Thereafter, a specific band of light is directed and radiated toward the target area of the subject.

The two main nanotechnology-based phototherapies for tumor ablation are photodynamic therapy (PDT) and photothermal therapy (PTT) (9). PDT is a two-stage treatment combining a photosensitizing chemical substance with activation by a specific wavelength and energy of light. This photosensitizer activation, performed in the presence of oxygen, generates singlet oxygen molecules that are highly reactive, extremely toxic, and known to cause damage to the tumor (12–14). In contrast, PTT is based on photothermal agents (PTAs) that convert near-infrared (NIR) light to vibrational energy (heat) (15). The targeted cancer cells are labeled by biocompatible PTAs with high photothermal conversion efficiency and stability, these being properties that permit the utilization of light in the near-infrared wavelengths (λ ≈ 650 - 1064 nm) (16). These long wavelengths are suitable for deep penetration into biological tissues (up to a few centimeters) due to their insignificant absorbance by water (17). Nevertheless, PDT and PTT have limited efficiency in cancer treatment (18). The shortcomings and drawbacks of these therapies include short chemical stability, poor tissue penetration, low oxygen, the short life and diffusion distance of ROS, and thermal damage to nearby healthy tissue (18–20). Optimization of the treatment parameters can enhance the efficiency of the treatment and allow these drawbacks to be overcome.

Synergistic strategies are becoming necessary to improve the treatment efficiency of PDT and PTT (21–23). Obstacles may be overcome by adopting a synergistic approach in which the disadvantages of one therapy are mitigated by the advantages of another. Under many circumstances, combining distinct therapeutic approaches in this way does not merely provide a simple supplementary effect, but in fact has a synergistic effect (24, 25). Thus, a thorough investigation should be made of the potential for a synergistic effect of PDT and PTT, rather than treating them as a simple combination of therapeutic approaches. The optimal equilibrium between these therapeutic approaches should be identified computationally in order to provide personalized and productive cancer treatment. Currently, there is no computational methodology that combines these phototherapies in an optimized and customized manner.

A major challenge in RT and particularly in phototherapy is the ability to predict a clinical radiation dose and outcome for a given treatment setup (26). Adjusting the treatment guidelines, such as by estimating the effective dose, reducing acute/late toxicity, and estimating conventional fractionation, is integral to the success of the treatment in terms of patient outcome. Currently, RT is offered based on the patient’s type of cancer, its location, volume, and proximity to normal tissue, and the patient’s general health and medical history (27, 28). After confirming the suitability of the treatment, the treating physicians attempt to identify the set of radiation properties, including radiation frequency, intensity, duration, and angle, that will optimize its effect on the tumor tissue (29). Nonetheless, personalized phototherapy combined with nanotechnology has not been adopted in a clinical setting (10). At present, there are no computational methods that incorporate an optimization criterion covering all aspects of treatment and offer a framework for prediction of a nanotechnology-based phototherapy cancer treatment (PDT combined with PTT).

In this article, we propose a framework for data analysis that may assist clinicians in designing a phototherapy treatment plan, with a corresponding setup configuration and specific laser radiation parameters, based on a desired treatment goal. The framework is based on machine learning techniques that enhance the treatment’s efficacy and the specificity with which it targets cancer cells. We focus in the present study, without loss of generality, on optimization of the laser radiation intensity and duration used in PDT and PTT, both separately and in combination. We derive mathematical models that include the condition of affected cells during and after the treatment; these derivations are followed by an evaluation of the gain provided by combined treatment. We propose three prediction models that enable the selection of an optimal set of parameters and enhance the overall treatment efficiency while preserving certain constraints. To validate the proposed model, we performed a set of *in vitro* experiments in which PDT, PTT, and a combination of the two were administered with various parameter setting conditions for each treatment. We conduct analysis of post- treatment cell death as a proxy for the treatment’s effectiveness.

The main objective of this work was to establish a precision medicine platform that predicts a treatment outcome when phototherapy and nanotechnology are combined. The work makes four main contributions: 1) formulation of a first step toward a decision support framework for clinicians to help them determine parameter optimization in combined laser radiation and nanotechnology- based treatment; 2) verification of the proposed framework using three predictive models based on machine learning that separately determine treatment parameters for PDT and PTT in order to achieve desired level of treatment efficiency while minimizing laser power and duration; 3) derivation of a quantitative model that aids in predicting the likelihood of cancer cell death based on post- treatment permeability measurements; and 4) derivation of a lower bound for the efficiency of combined PDT and PTT therapies based on the prior distributions of the estimated efficiency of each treatment separately .

## 2 Materials and methods

### 2.1 Materials

#### 2.1.1 Experimental setup

**Figure 1:**
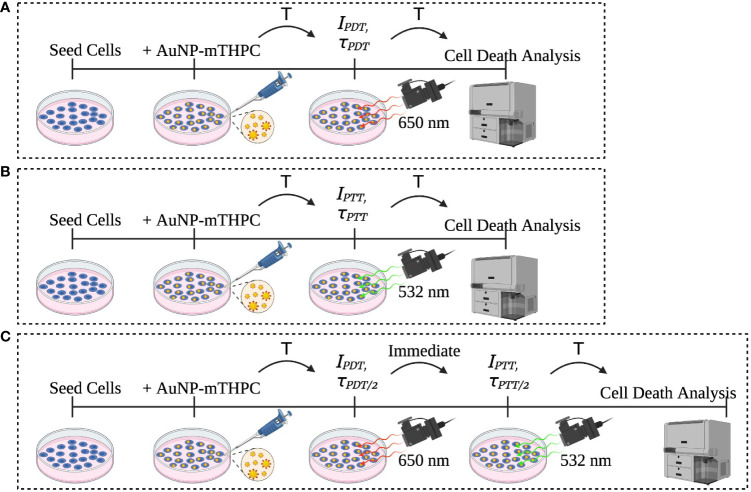
Description of the experiment sets: **(A)** PDT, **(B)** PTT, and **(C)** the combined PDT and PTT experiments.

Human neuroblastoma cells SH-SY5Y (ATCC) were incubated in a 24-well culture plate at a density of 1 X 10^5^ per well. After 24 h of incubation, the cells were treated with 1.2 μM AuNP-mTHPC in serum-free media and co-incubated for another 24 h without light interference. The characterization of this nanocomplex has been described previously (25). Subsequently, the cells were exposed to 650 nm laser (PDT), 532 nm laser (PTT), or a combination of PDT and PTT. After laser radiation, the cells were incubated at 37°C for 24 h (**Figure 1**). Following this procedure, all cells were collected from the dish and propidium iodide (PI) was added; samples were next incubated in darkness for 5 minutes at room temperature and then analyzed using flow cytometry (BD LSRFortessa™).

**Figure 2:**
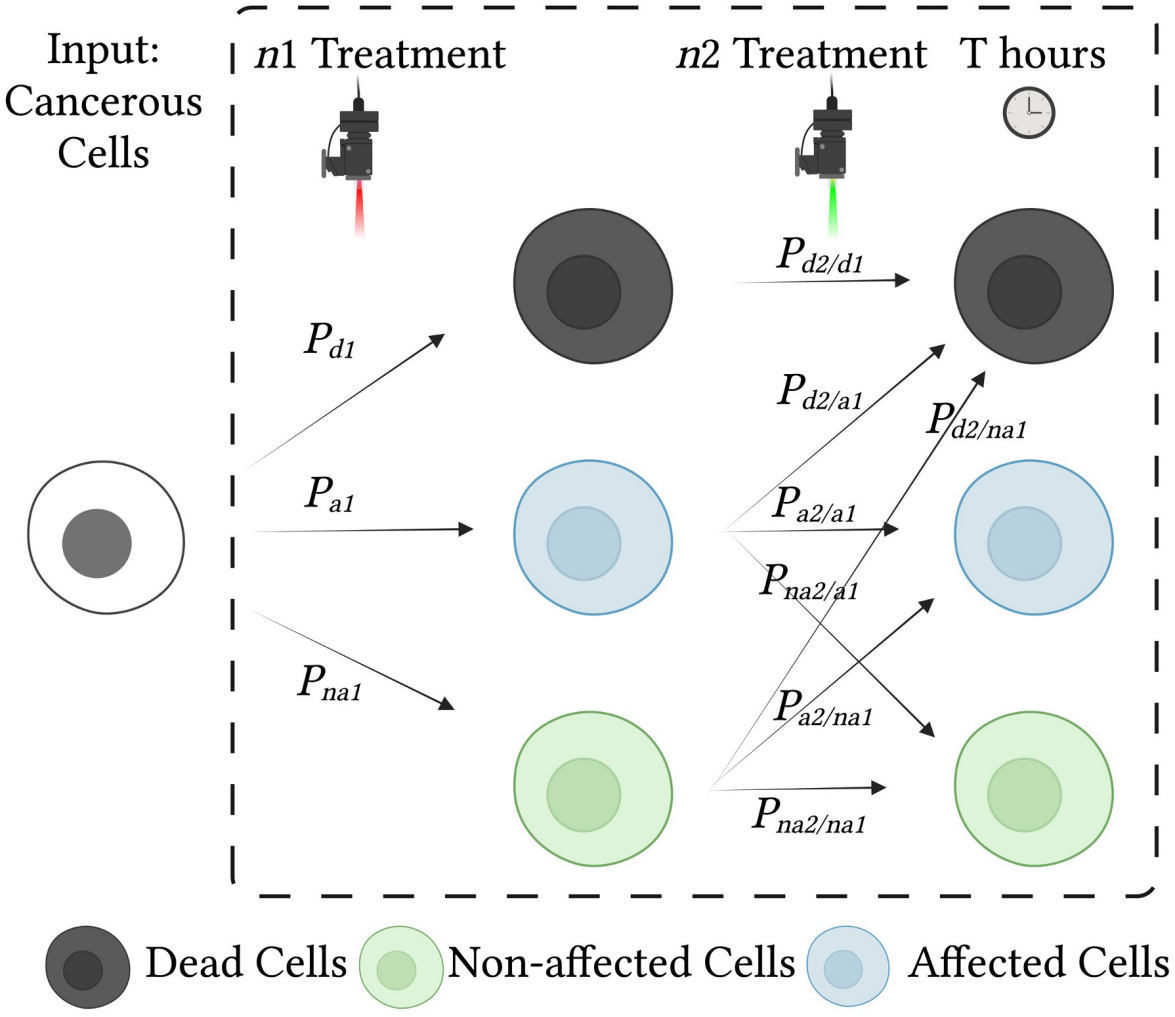
Effects of treatment on cells and probabilities of each cell state. Cancerous cells are treated with two consecutive treatments, *n*1 and *n*2. Each treatment influences the condition of the cells and may result in cell death, cell damage (affected cells), or no change (unaffected cells). To analyze the effect of the combined treatment, we use a state diagram with conditional probabilities.

For the cell death analysis, we used the standard FACS software based on cell size and fluorescent intensity (30). Fractional cells and doublet cells were filtered out as artifacts and excluded. Clustering of cells into live and dead clusters was derived *via* a manual gating process. Data were processed and analyzed using MATLAB software (MATLAB version R2021a, The MathWorks, Inc). **Figure 2** shows the PDT, PTT, and combined PDT and PTT experiments.

#### 2.1.2 Sets of experiments

We conducted separate sets of experiments for training and testing of the prediction model. To obtain training data for the model, we carried out two main sets of experiments for PDT and PTT (25). For each set, we used laser radiation intensities of 1, 6, 15, and 30 mW/cm^2^ and treatment durations of 1, 4, and 8 minutes, for a total of 12 experimental configurations, each of which was implemented twice for replication purposes. This array of laser radiation parameters was designed to include extreme conditions that would result in unaffected cells and death of the entire set of the cells used in the experimental setup. For each experiment we had three controls: cells without any treatment, cells exposed to laser radiation only (with no NPs), and cells exposed to NPs only (without laser radiation).

To test the prediction model, we used an independent test dataset. This test set consisted of two subsets of data, the first consisting of data from three experiments in which each treatment was administered separately, and the second consisting of data from two in which the treatments were combined. This allowed us to examine the feasibility of predicting a lower bound for the efficiency of the combined treatment, over a total of eight test experiments. The test values for a tuple representing treatment duration and intensity, (τ, *I*_0_), were selected randomly from the range of values falling within the scope of the treatment; these selections were (2, 25), (6, 12), (7,18), which were used for both the PDT and the PTT treatments, generating a total of six datapoints for the test set.

The set of values used for laser radiation were selected in accordance with the relevant FDA regulations, which specify that Photofrin^®^ should be activated with laser light delivered through optical fibers with a cylindrical-diffuser end emitting 630-nm light at an intensity of 400 mW per cm (mW/cm) and energy of 50–300 J/cm per length of the diffuser (31).

To examine the effect of combined PDT and PTT treatment, we administered identical total treatment durations of 4 and 8 minutes for a fair comparison. For the first dataset, PDT and PTT were administered separately for a duration of 4 minutes in each case, and in the combined condition, each was administered for a duration of 2 minutes. For the second dataset, each treatment was administered separately for a duration of 8 minutes, and in the combined condition, each was administered for 4 minutes. For each of the two sets, laser radiation intensity was set to 6 and 15 mW/cm^2^ for PDT and PTT, respectively. Thus, the values of the parameter tuple (τ, *I*_0_) for the test datasets with combined PDT and PTT treatments were (4, 6), (4, 15), and (8, 6), (8, 15). These values were selected to produce similar treatment efficacies that would enable examination of the effect of the combined treatment without exceeding the maximum value of any parameter. We first applied PDT, followed by PTT; the order of application has been demonstrated to be approximately commutative.

### 2.2 Methods

#### 2.2.1 Modeling of combined laser radiation and nanotechnology-based phototherapy

In RT, an electromagnetic power source is concentrated to form a beam, which is radiated toward the target tissue. The radiated beam, *B*, can be defined (following 32) as:


(1) $$B\left(\theta ,f_{c},I_{0}\right)=a\left(\theta \right)I_{0}e^{2\pi f_{c}t}.$$

where $I_{0}^{1/2}e^{2\pi f_{c}t+\varphi }$ represents the electromagnetic waveform, *f_c_*, and *φ* are its frequency and phase, and α(*θ*) represents the spatial attenuation factor, which is a function of the beamforming technique and the electromagnetic waveform properties.

The beam is radiated in the desired direction with a pre-determined intensity, frequency, and duration (*τ*) that alter the properties of the affected tissues (3, 33). Thus, the spatial attenuation factor can determine the spatial selectivity of the radiation treatment.

The cellular properties of cancer cells differ from those of normal cells in several respects, such as membrane permeability, cellular morphology, and gene expression (34). A nanotechnology phototherapy design as described by Haimov etal. (14) or Raj etal. (35) aims to exploit the difference in permeability to enable NPs to enter the cancer cells exclusively. This targeted nanotechnology approach is dependent on tissue composition, endothelial cell junctions, tumor location, and the characteristics of the NPs (20, 36, 37).

Overall treatment selectivity is affected by radiation selectivity, which is determined by the beam spatial attenuation, *a*(*θ*)), and by nanotechnology selectivity (38). Thus, the overall selectivity is determined by the nanomaterial, the cell membrane permeability, and the properties of the radiation:


(2) $$\;S_{n}=\text{fS(}m_{n},C_{p},B(\theta ,f_{c},I_{0})$$

Where *m_n_* represents the set of relevant NP properties such as size, material, and shape; *C_p_* represents the permeability-related properties of the cancerous and normal cells in the tissue undergoing treatment; and *B*(*θ,f_c_*) represents the properties of the radiation targeting the selected area.

**Figure 3:**
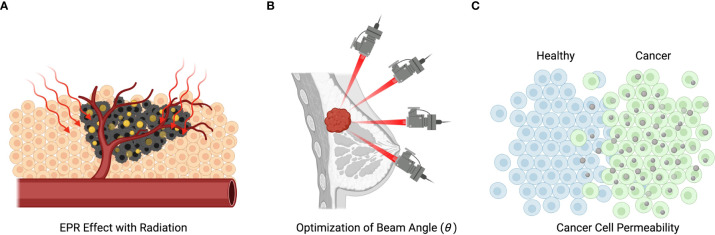
Mechanisms of radiation and nanotechnology-based treatment. **(A)** Illustration of nanoparticles entering the metastatic cells and being subsequently activated by radiation. **(B)** Illustration of the spatial selectivity of traditional radiotherapy, which is spatially selective, but can cause death of not metastatic cells. **(C)** Demonstration of how metastatic selectivity together with spatial selectivity can enhance treatment efficacy.

**Figure 3B** illustrates the spatial sensitivity of traditional radiotherapy aiming to minimize damage to normal cells. **Figure 3C** illustrates how treatment selectivity for cancer cells over normal cells can be used together with spatial selectivity to enhance the overall efficacy of a combined treatment.

A prerequisite for personalized cancer treatment is the ability to optimize treatment efficacy while preserving certain constraints. Here, the major criterion for treatment efficiency is the ability to terminate cancer cells while sparing normal cells from any harm. The treatment can be administered under certain safety constraints to ensure that radiation will not cause irreversible damage to non-targeted tissues (39); these constraints include NP biocompatibility (40, 41) and reduced treatment duration. In addition to safety considerations, treatment costs and computational difficulty can also be considered.

We define the response to treatment *n*, with treatment conditions *P_n_*, as the cell death rate as sampled *T* hours after the beginning of the experiment; this can be represented by the expression $D_{P_{n}}^{n,\;T}$ . The cell death rate represents the probability that an individual cell within a sampled population will die, and its possible values range from 0 to 1. The treatment conditions, for example, might consist of laser radiation intensity (mW/cm^2^) and duration (min).

An efficiency criterion for maximization in laser radiation-based cancer treatment is:


(3) $$L_{P_{n}}^{n,T}=\frac{D_{P_{n}}^{n,T,\text{cancerous }cells}}{D_{P_{n}}^{n,T,normal\;cells}}-L_{P_{n}}^{\tau }+L_{P_{n}}^{safety}$$

where, $D_{P_{n}}^{n,T,\text{cancerous }cells}$ and $D_{P_{n}}^{n,T,normal\;cells}$ represent the total death rates among the targeted cancerous and normal cells, respectively; $L_{P_{n}}^{\tau }$ is a clinical cost represented as a function of the treatment duration; and $L_{P_{n}}^{safety}$ is a safety cost, usually a function of the laser radiation intensity and the nano-material component.

This criterion implies that the ratio $\frac{D_{P_{n}}^{n,T,\text{cancerous }cells}}{D_{P_{n}}^{n,T,normal\;cells}}$ is the treatment selectivity, $S_{P_{n}}^{n,T}$ , which needs to be maximized while preserving feasible treatment duration and safe radiation conditions. Treatment latency, which is a component of the treatment cost, must also be minimized, while treatment safety must be maximized, or fall within the boundaries of the FDA regulations (31).

#### 2.2.2 Estimating target cell death under a single treatment (PDT or PTT)

Death rate is a non-linear function that is determined by the treatment conditions, laser radiation intensity, and duration (42). To estimate the death rate of targeted and non-targeted cells, we need to calculate the death rate as a function of the treatment condition and compare it to a reference baseline. The cell death rate compared to the reference baseline can be defined as:


(4) $$D_{P_{n}}^{n,T}=f_{P_{n}}^{n,T}\left(D^{0}\right)-D_{P_{control}}^{T}$$

where *D* ^0^ is the initial death rate of the cells, $f_{P_{n}}^{n,T}$ is a function of the *n*th set of treatment conditions *P_n_*, sampled at time *T*, and $D_{P_{control}}^{T}$ is the control death rate in the absence of the treatment, also sampled at time *T*, which is used as a reference.

Thus, the treatment success rate as a function of the treatment parameters *I* (laser radiation intensity) and *τ* (laser radiation duration), according to a sample taken after 24 hours from the experiment onset, is defined as follows for the PDT and PTT treatment conditions, respectively:


(5) $$D_{I,\tau }^{PDT,24}=F_{I,\tau }^{PDT,24}\left(D^{0}\right)-D_{P_{control}}^{T},\;D_{I,\tau }^{PTT,24}=F_{I,\tau }^{PTT,24}\left(D^{0}\right)-D_{P_{control}}^{T}$$

#### 2.2.3 Dual treatment design (PDT and PTT)

In the case of the dual PDT and PTT treatment design, death rate is assessed as follows:


(6) $$D_{P_{n_{1}},P_{n_{2}}}^{n_{1}+n_{2},T}=f_{P_{n_{2}}}^{n_{2},T}\left(f_{P_{n_{1}}}^{n_{1},T}\left(D^{0}\right)\right)-D_{P_{control}}^{T}$$

For example, in a case in which PDT and then PTT treatments are administered, and the tissues sampled after 24 hours, with the treatment parameters *I*_*PDT*_,*τ*_*PDT*_ , and *I*_*PTT*_,*τ*_*PTT*_ , we would compute the following death rate:


(7) $$D_{I,\tau \;}^{PDT+PTT,T}=f_{I_{PTT},\tau_{PTT}}^{\text{PTT},T}\left(f_{I_{PDT},\tau_{PDT}}^{\text{PDT},T}\left(D^{0}\right)\right)-D_{P_{control}}^{T}$$

For an unbiased comparison between any single treatment and the combined effect, we equate the laser radiation intensity and duration used in the combined PDT and PTT treatment to that of an individual PDT or PTT treatment,*τ_PTT_* or *τ_PDT_*.

#### 2.2.4 Optimization criterion

To determine the optimal efficiency of a given treatment , we need to solve for the criterion in (3), as a function of the treatment conditions under the treatment latency and safety constraints. We can do this using the *L2*-norm loss function, while minimizing the difference between treatment sensitivity and the desired sensitivity goal, and with the addition of the safety and duration losses as constraints. The criterion for identification of the optimal treatment conditions for the *n*th treatment, after *T* minutes, thus becomes:


(8) $$L_{P_{n}}^{n,T}=E{\left(S_{desired}^{T}-S_{P_{n}}^{n,T}\right)}^{2}s.t.\;\;L_{P_{n}}^{\tau },L_{P_{n}}^{safety}$$

Solving for this criterion can be cumbersome. We can assume, without loss of generality, that the death rate of the normal cells, $D_{P_{n}}^{n,T,normal\;cells}$ , is both low and relatively constant, and can therefore be neglected. We further assume that the NPs employed in application of the treatment are biocompatible. Accordingly, the only tunable safety parameter is that relating to laser radiation. The treatment optimization criterion then becomes:


(9) $$L\left(I,\tau \right)=E{\left(D_{desired}^{n,T,\;\text{cancerous }\;cells}-D_{P_{n}}^{n,T,\text{cancerous }\;cells}\right)}^{2}s.t.\;\tau <\tau_{max},\;I_{0}<I_{0max}$$

where *τ*<*τ_max_* represents the treatment latency constraint and *I*_0_<*I*_0,_ *_max_* represents the safety constraint, with *I*_0,_ *_max_* being the maximum laser radiation.

#### 2.2.5 Identifying treatment parameters to maximize treatment efficiency

The criterion in (9) can be used to identify the optimal treatment parameters. We assume without loss of generality that the treatment parameters consist of laser radiation intensity and treatment duration. Thus, the solution to (9) can be obtained by solving for the following criterion:


(10) $$\left[\hat{I},\hat{\tau }\right]=argmin_{I,\tau }E{\left(D_{desired}^{n,T,\text{cancerous }cells}-D_{I,\tau }^{n,T,\text{cancerous }cells}\right)}^{2}s.t.\tau <\tau_{max},\;I_{0}<I_{0max}$$

where $D_{desired}^{n,T,Mcells}$ is the desired death rate of the cancerous cells and $D_{I,\tau }^{n,T,Mcells}$ is the measured death rate as a function of the treatment parameters (laser radiation duration and intensity).

The solution implies that the treatment parameters should be identified while the constraints are preserved. To solve (9), we can use predictive models based on training datasets (18) that capture the statistical outcomes of a range of parameter settings. Although the problem is non-linear, we can exploit high- order polynomials, as in Blumrosen etal. (39). Our objective is to predict continuous cell death rate values, rather than predicting a predetermined set of classes. Therefore, continuous methods like regression and interpolation are appropriate. Non-linear regression can include many coefficients, which means that they can suffer from overfitting and cause estimation errors to accumulate. Accordingly, we also propose adopting an approach to fitting using analytical functions that preserve the asymptotic conditions of the solution.

#### 2.2.6 Single treatment effect analysis

If the influence of the two treatments on the viability of cells is independent and there are no live but affected (i.e., partially damaged) cells, their combined effect in terms of efficiency can be defined as the additive sum of the two efficiencies. Under a realistic set of model assumptions, we assume the following: 1) there are some cells, referred to as “affected cells”, that incur partial damage; 2) a portion of the cells with partial damage will recover naturally; 3) another portion of the cells with partial damage will accumulate further damage under the effect of the other treatment, which is likely to contribute to the cell death rate.

We model the probability of cell death after treatment as *p_D_*, which can be estimated as a function of the experimental conditions. The cell death rate associated with a particular experimental setup can be measured based on the fluorescent intensity of the cells present in the training data, as this variable attests to the cells’ condition (43, 44). Specifically, a signal indicating high fluorescent intensity can be taken to indicate reduced cell viability, since it means that membrane permeability is greater and consequently the cell has a higher probability of dying.

Traditionally, cells have two possible conditions: live or dead. In practical terms, in our work, we measure the probability of cell death by examining the population. We can determine the probability of cell death if the population statistic exceeds a threshold of *D_TH_* for the value that ensures the cell’s death:


(11) $$p\left(D_{P_{n}}^{n,T}\right)=F_{D}(D_{P_{n}}^{n,T}>D_{TH})$$

where *F_D_* is the cumulative function of the probability distribution, and the probability that a cell will be unaffected by the treatment is $1-p\left(D_{P_{n}}^{n,T}\right)$ . *D_TH_* is determined in such a way as to ensure that a desired sensitivity and specificity are achieved in this classification, e.g., specificity of 0.95. The results need to be verified either by inspecting the properties of the cells or by tracing the sampled cells for a long period.

In this work, to model the intermediate state, we define three cell states: unaffected, affected (partially damaged), and dead. Detection of damaged cells is challenging (45), but cell damage following this type of treatment results in alterations to overall cell volume, cell circularity, and membrane integrity.

**Figure 4:**
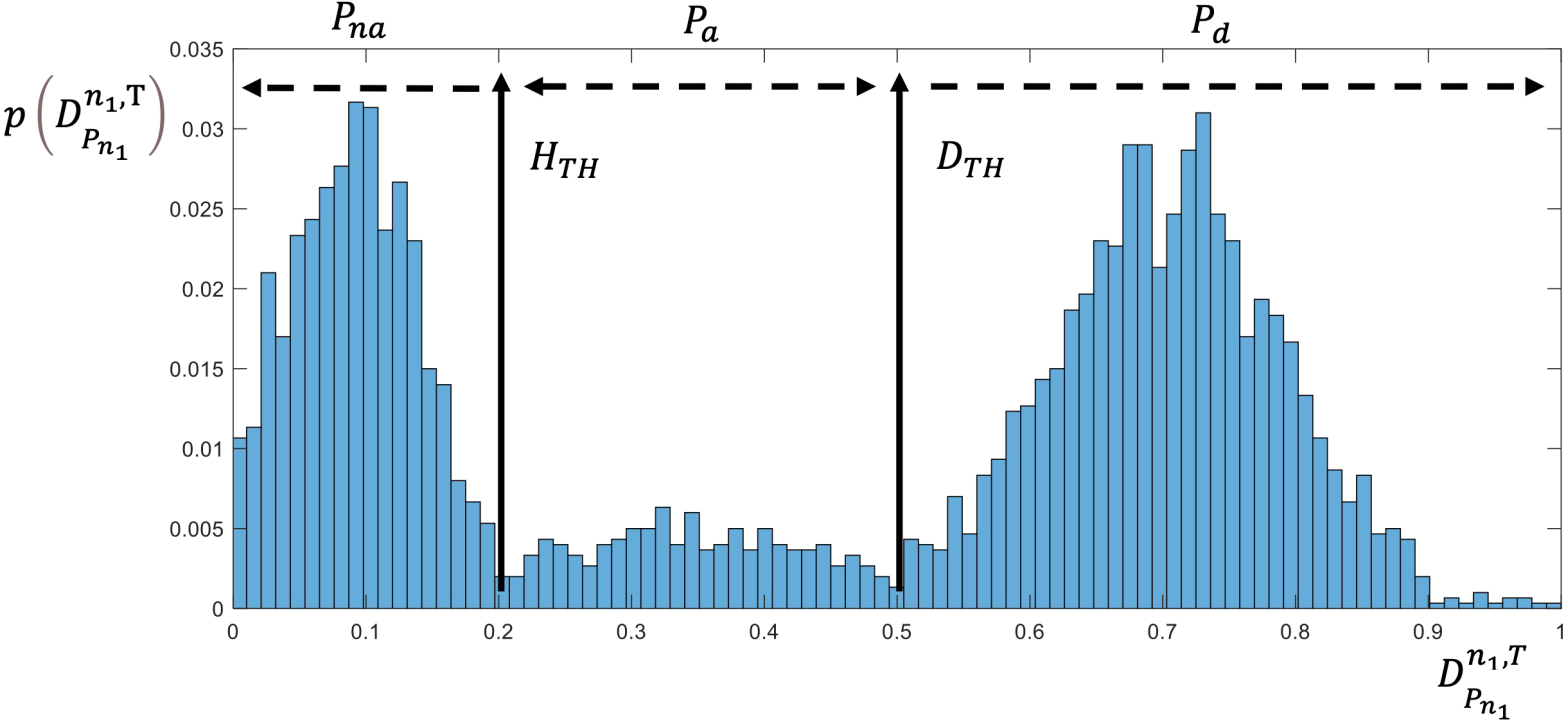
Cell state as derived by the death rate probability.

To estimate the condition of the cells, and specifically to estimate the death rate, $D_{P_{n}}^{n,T}$ , an accepted method is to observe two main features: cell size and membrane permeability. Normal cells will retain their size and exclude fluorescent binding dyes, whereas dead or damaged cells are drastically smaller and contain permeable membranes that allow fluorescent materials such as propidium iodide (PI) to enter unimpeded (43). The clustering of cell states is a k-dimensional problem, where k is the number of features. A machine learning algorithm can be used to locate the decision boundaries that maximize the selectivity and specificity of the clustering algorithm. Under the assumption that some of the cell state parameters are continuous, such as the correlation of the fluorescence signal with cell death, the problem can be reduced to a single dimension with two thresholds based on the probability of cell death within the population. As an example, the clustering of cells into the three conditions of unaffected, affected (partially damaged), or dead can then be approximated by:


(12) $$p\left(s\right)=\left|\begin{array}{c} F_{D}\left(D_{P_{n}}^{n,T}>D_{TH}\right)\;\;\;\;\;\;\;\;\;\;\;\;\;\;\;\;\;\;\;\;P_{d}\\ \;F_{D}\left(D_{P_{n}}^{n,T}>H_{TH}\right)+\;F_{p_{D}}\left(D_{P_{n}}^{n,T}<D_{TH}\right)\;P_{a}\;\\ \;F_{D}\left(D_{P_{n}}^{n,T}<H_{TH}\right)\;\;\;\;\;\;\;\;\;\;\;\;\;\;\;\;\;\;\;\;P_{na}\; \end{array}\right|$$

where *P_d_*, *P_a_* and *P_na_*, are the probabilities of death, being affected (partially damaged), and being unaffected (normal), respectively, and the thresholds for death and being unaffected (*D_TH_*, and *H_TH_*) are selected for the desired sensitivity and specificity (46). **Figure 4** illustrates the decision boundaries used to classify the conditions on the basis of the cell death probability density function, $p(D_{P_{n}}^{n,T}$ ), with classification carried out on a simulated randomized dataset with mean values of 0.1, 0.3, and 0.7 for the proportion of unaffected, affected, and dead cells, respectively.

#### 2.2.7 Effect of combined treatment

The combined effect of multiple cancer treatments is of great importance in cancer therapy (47). The primary consideration is that the concatenation of different treatments can increase the level of accumulated damage to the targeted cells in a diverse manner, thereby improving the overall effectiveness of treatment (25). Secondarily, the two treatment effects described in (6) combine with one another in a non-linear fashion, which makes it cumbersome to optimize this combined treatment based on separate optimization of each individual treatment. Consequently, non-linear parameter optimization can enhance performance in a desirable way, in a similar manner to hyperparameter configuration of non-linear neural networks (48). We derive a statistical model of this combined effect below.

The presence of damaged cells induces a statistical dependency between treatments (whether they involve repetition of the same treatment or administration of a different treatment), as the probability that damaged cells will recover or die depends on the prior probability. The probability of death after consecutive treatments becomes:


(13) $$p\left(D_{P_{n_{1}},P_{n_{2}}}^{n_{1}+n_{2},T}\right)=P_{d_{1}}\left(D_{P_{n_{1}}}^{n_{1},T}\right)P_{d_{2}/d_{1}}\left(D_{P_{n_{2}}}^{n_{2},T}\right)+P_{a_{1}}P_{d_{2}/a_{1}}\left(D_{P_{n_{2}}}^{n_{2},T}\right)+P_{na_{1}}P_{d_{2}/na_{1}}\left(D_{P_{n_{2}}}^{n_{2},T}\right)$$

The term $P_{d_{2}/d_{1}}$ is equal to 1 since dead cells remain dead.

A combined treatment gain can be defined as the relative gain occurring as a result of two consecutive different treatments compared to that occurring as a result of consecutive applications of the same treatment (24, 49, 50). Thus, the gain is:


(14) $$S_{G}(n_{1},n_{2}|n_{1},n_{1})=p\left(D_{P_{n_{1}},P_{n_{2}}}^{n_{1}+n_{2},T}\right)-p\left(D_{P_{n_{1}},P_{n_{1}}}^{n_{1}+n_{1},T}\right)$$

The combined effect can also be denoted as *S_G_* (*n*_1_,*n*_2_|*n*_1_).

A value of *S_G_* (*n*_1_) > 0 indicates a synergistic result. This implies that application of the two processes has a higher efficiency than the option of repeating the same treatment. When *S_G_* (*n*_1_) = 0, there is no synergetic gain; instead, the gain between treatments is additive. Finally, when *S_G_* (*n*_1_)< 0, the efficiency is higher with repetition of treatment *n*_1_ ; consequently, the gain is of a type referred to as antagonism.

Substituting the terms in (13) into (14), we derive the combined gain as follows:


(15) $$S_{G}\left(n_{1},n_{1}\right)=P_{a_{1}}P_{d_{2}/a_{1}}\left(D_{P_{n_{2}}}^{n_{2},T}\right)+P_{na_{1}}P_{d_{2}/na_{1}}\left(D_{P_{n_{2}}}^{n_{2},T}\right)-P_{a_{1}}P_{d_{2}/a_{1}}\left(D_{P_{n_{1}}}^{n_{1},T}\right)-P_{na_{1}}P_{d_{2}/na_{1}}\left(D_{P_{n_{2}}}^{n_{2},T}\right)$$

The combined gain can be further separated into two components, covering the effect of the combined treatment on damaged cells and unaffected cells:


(16) $$S_{G}\left(n_{1},n_{2}|n_{1}\right)=S_{\text{G}}^{a_{1}}+S_{\text{G}}^{na_{1}}\;$$

where $S_{G}^{a_{1}}$ and $S_{G}^{na_{1}}$ represent the combined gain in relation to affected (partially damaged) cells and unaffected (normal) cells, respectively, $P_{a_{1}}\left(P_{d_{2}/a_{1}}^{n_{2}}-P_{d_{2}/a_{1}}^{n_{1}}\right),P_{na_{1}}\left(P_{d_{2}/a_{1}}^{n_{2}}-P_{d_{2}/a_{1}}^{n_{1}}\right)$ .

Following the initial treatment, affected cells have incurred preliminary damage, which reduces their resistance to the second treatment. The combined gain depends on the treatment condition and on the details of the experimental setup, including cell type, NP characteristics, and the accuracy with which cell states are measured. Quantification of the effects of the combined treatments can be used to estimate the combined gain; this entails considering the internal states of the cells after the initial treatment. The probability of cell death in unaffected cells is governed by two factors: death may occur either as a result of treatment or through spontaneous natural death. We assume subtraction of the natural cell death rate in the control experiment in (4), hence it can be neglected. **Figure 4** illustrates the cell state distribution after a single treatment and dual consecutive treatments.

#### 2.2.8 Prediction of the combined treatment efficiency

To predict the efficiency of the combined treatment, we need to estimate the distributions for each treatment. An alternative method is to use the prediction model to make estimates based on measurements taken following different components of the combined treatment.

Excluding deaths among normal cells and the presence of damaged cells (since these are not clustered as dead cells) we derive a lower bound for the probability of cell death under the combined treatment:


(17) $$p\left(treatment\;effect\;independent\right)=P_{d_{1}}\left(D_{P_{n_{1}}}^{n_{1},T}\right)+\left(1-P_{d_{1}}\left(D_{P_{n_{1}}}^{n_{1},T}\right)\right)P_{d_{2}}\left(D_{P_{n_{2}}}^{n_{2},T}\right)$$

A further lower bound can be established based on the assumption that there is no synergetic effect of the treatments, and we neglect deaths occurring among unaffected cells (natural death) and damaged cells. Accordingly, the two treatments each have an independent effect based on the unique influence of each on the viability of cells. The lower bound then becomes:


(18) $$B_{L}\left(D_{P_{n_{1}},P_{n_{2}}}^{n_{1}+n_{2},T}\right)=P_{d_{1}}\left(D_{P_{n_{1}}}^{n_{1},T}\right)+\left(1-P_{d_{1}}\left(D_{P_{n_{1}}}^{n_{1},T}\right)\right)P_{d_{2}}\left(D_{P_{n_{2}}}^{n_{2},T}\right)$$

The lower bound in a case in which the predicted outcome of the first treatment alone is $P_{d_{1}}\left(D_{P_{n_{1}}}^{n_{1},T}\right)=0.6$ , and that of the second treatment is $P_{d_{2}}\left(D_{P_{n_{2}}}^{n_{2},T}\right)=0.7$ , would be $B_{L}\left(D_{P_{n_{1}},P_{n_{2}}}^{n_{1}+n_{2},T}\right)=0.6+\left(1-0.6\right)\times 0.7=0.84$ . If the result of combined treatment is a higher death rate than the lower bound, this indicates a synergetic gain: for example, experimental results demonstrating a death rate of 0.94 would indicate a synergetic gain of 0.1, or 12%.

## 3 Results

### 3.1 Pre-processing and artifact removal

Pre-processing was performed on the raw data on cells’ fluorescent intensity, which was measured as an indicator of the rates of cell membrane damage and cell death. First, we computed the normalized level of fluorescence in comparison to a baseline reference; this was taken to represent the probability of cell death . Subsequently, we excluded artifacts on the basis of extreme cell size values that would be unrepresentative of cells. For appropriate artifact removal we used the standard protocol for flow cytometry analysis. Fractional cells and doublet cells were treated as artifacts and excluded. The clustering of cells into live and dead clusters was derived in accordance with a manual gating process in which the size of the cells was also used as a criterion for removal of artifacts arising from fractional cells and doublets, as shown in **Figure 4**.

Flow cytometry analysis typically begins with the creation of gates and regions to quantify cells of interest. Specifically, forward and side scatter density plots (the former denoting cell size and the latter detailing the granularity of the cell) are used for the identification of distinct cells in a population and to exclude debris. Nonetheless, these FSC and SSC values are merely indications of size and granularity, since they are dependent on the nature of the sample, light refraction, sheath fluid, and laser wavelength. Debris and doublet cells can drastically influence this type of analysis and may lead to mistaken conclusions. Thus, these cells were treated as artifacts and excluded from our analysis (51).

Following this process, we derived from the fluorescent intensity the probability of cell death in a similar manner to Verschoor etal. (30). We then subtracted the control samples from the experimental data. This provided the ability to visualize and demonstrate the combined effect of the NPs and laser radiation. Specifically, we subtracted the mean cell death rate observed in both control experiments (cells treated with NPs only and cells treated with laser radiation only).

### 3.2 Raw data representation

**Figure 5:**
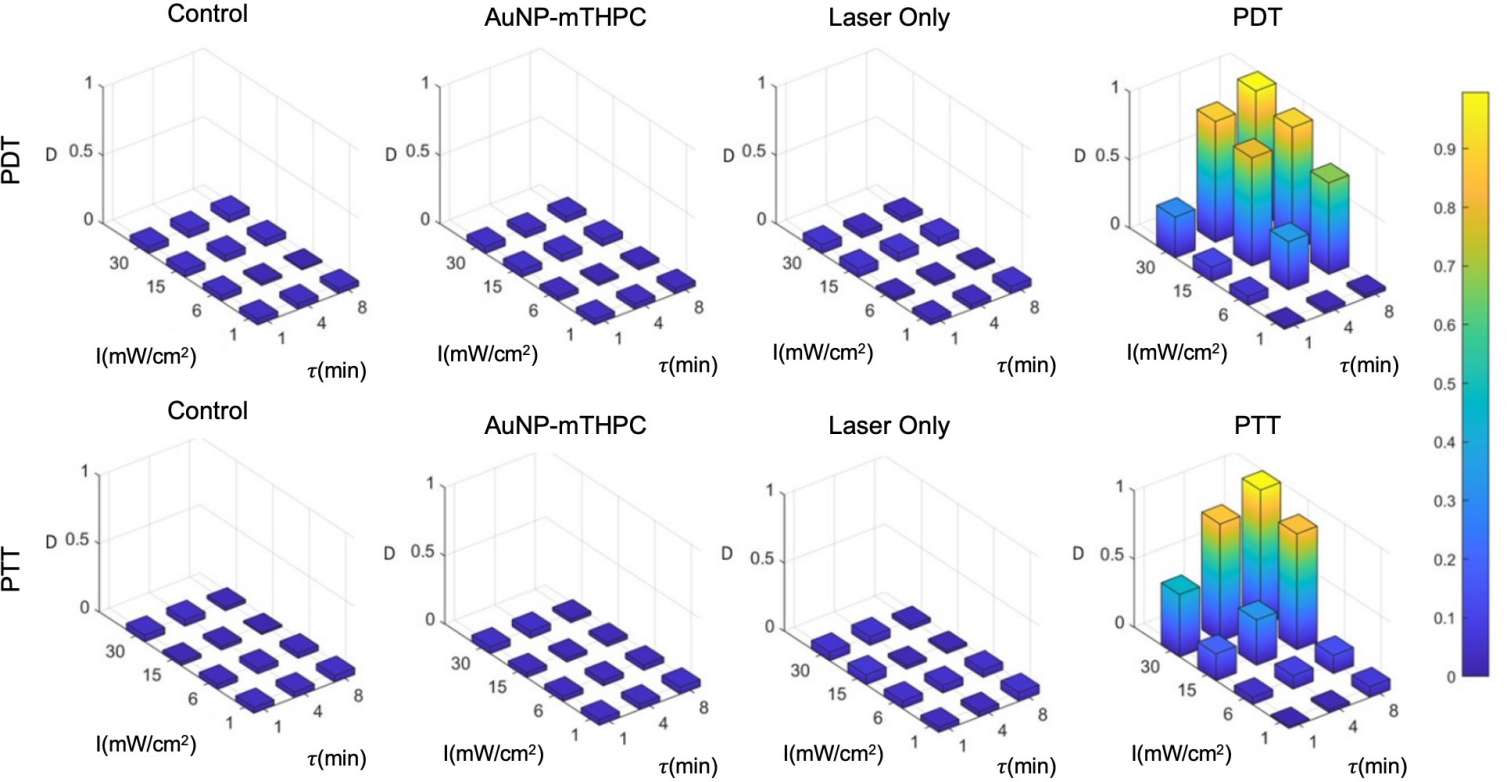
PDT and PTT treatment for different feasible treatment durations (min) and intensities (mW/cm^2^).

The result of the calibration experiment is shown in **Figure 5**. It can be seen in this figure that a similar death rate (4.0±2.5%) was observed in all control groups after 24 hours, while the death rates in treated samples ranged from 3% to 99%. For a single treatment, in practice, we may opt to use the highest intensity and longest feasible duration: for example, 30 mW/cm^2^ and 8 minutes. However, since the objective of the present study was to establish the incremental value of the combined treatment, we employed a set of parameters that would induce intermediate rates of cell death, creating the potential for gain as a result of the combination of PDT and PTT treatments.

Based on the results, we hypothesized that the death rate was elevated in cases of excessive intensities of over 15 mW/cm^2^ and treatment durations above 4 minutes. At the low end of the range of laser intensities and durations, the death rate was much lower, with little effect exerted on the sampled cells. We further observed that there was no substantial death rate in any of the controls (4.0±2.5%). Finally, we also observed that PDT produced stronger effects on cells at a lower duration in comparison to PTT; this might be attributable to the time required for NPs to heat up (**Figure 5**), which was around 90 seconds in our setup. Therefore, for the test data, we selected intermediate laser radiation values of 6 mW/cm^2^ for 4 minutes and 15 mW/cm^2^ for 4 minutes for the PDT and PTT treatments, respectively.

### 3.3 Treatment efficacy prediction models

Machine learning prediction models, used to approximate the efficiency of a given treatment, are essential in many clinical applications today (52). We compared three common prediction models: regression, interpolation, and analytical function fitting.

For the regression model, we used the LOWESS (Locally Weighted Scatterplot Smoothing) method, which is a non-parametric linear regression method. The LOWESS method works locally by using a sliding window in which each smoothed value is determined by its neighboring data points within a certain defined distance. We configured the sliding window span at 28% and used a robust weight function to combat the influence of outliers. For the interpolation model, we used thin-plate spline interpolation, in which the values lying between each training datapoint are interpolated and smoothed in accordance with a thin-plate spline shape. For the analytical function model, we used a second-degree polynomial fitting all data points.

**Figure 6:**
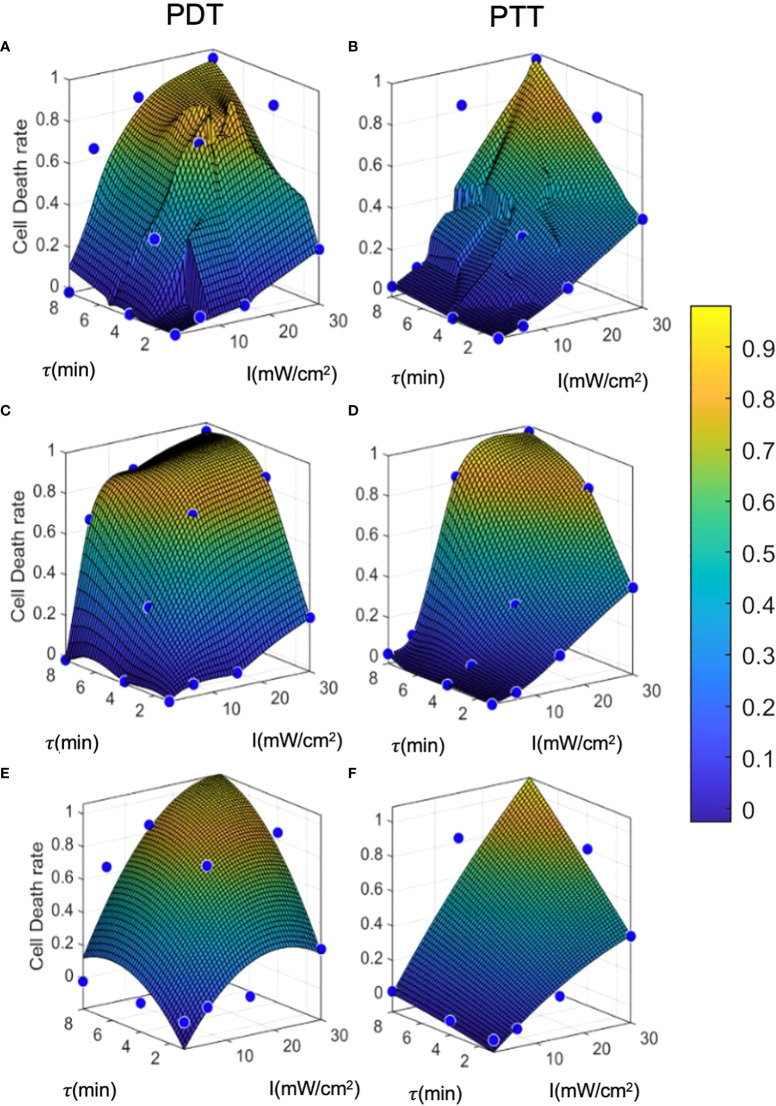
Prediction model curves. **(A-F)** illustrate the curves generated by prediction models based on regression, interpolation, and analytical function fitting for PDT and PTT, respectively. Blue points represent the mean values of training datapoints.

**Figure 6** displays the results of each of the three prediction models for the PDT and PTT treatments. For all prediction curves, the treatment efficiency (cell death rate) is positively proportional to the laser radiation intensity and duration. However, the relationship appears to be less linear in the case of PDT compared to PTT. For all prediction models, we observe that the PDT model produces a larger region of effective treatment. We can see that the efficiency of PDT treatment becomes greater in comparison to that of PTT with diminishing treatment durations. This can be explained by the time that is required for NPs to heat up (41, 42, 49).

The linear regression results for both PDT and PTT, illustrated in **Figures 6A** and **6B**, appear to be highly local, and some training datapoints seem to have high residual error values. This reflects the nature of regression models, where the accuracy of the model is directly affected by an inadequate sample size, causing local distortions. This results in relatively high standard deviation error of 4.8 ± 11 and 3.8 ± 10 percent. Nevertheless, the model performed well, with R^2 =^ 0.89 and 0.9 for PDT and PTT, respectively.

Under the interpolation model, illustrated for PDT and PTT in Fig 6c and 6d respectively, the training data points were retained as constraints and points between them were interpolated and smoothed using the thin-plate spline shape. Thus, the residual error at the training data points is 0 for both prediction models, with R^2 =^ 1. Predictions for regions between the points appear to be smoother and to reflect physical behavior.

Lastly, the analytical function model was selected to have a lower dimension rank of two, in the form of $D_{P_{n}}^{n,T}=a_{1}I_{0}^{2}+a_{2}I_{0}+a_{3}\tau^{2}+a_{4}\tau +a_{5}\tau I_{0}+a_{6}$ . The prediction was not local, allowing minimization of the fitting error for all training data points. The results of fitting were: *a*_1_=−0.001,*a*_2_=0.04,*a*_3_=−0.014,*a*_4_=0.170,*a*_5_=0.002,*a*_6_=−0.392; and *a*_1_=−0.0003 ,*a*_2_=0.02,*a*_3_=−0.0006 ,*a*_4_=0.018,*a*_5_=0.003,*a*_6_=−0.14 , for PDT and PTT, respectively:


(19) $$D_{\tau ,I_{0}}^{PDT,24}=-0.001I_{0}^{2}+0.04I_{0}-0.014\tau^{2}+0.17\tau +0.002\tau I_{0}-0.392$$

(20) $$D_{\tau ,I_{0}}^{PTT,24}=-0.0003I_{0}^{2}+0.02I_{0}-0.0006\tau^{2}+0.018\tau +0.003\tau I_{0}-0.14$$

The coefficients affirm that the linear components were more dominant in the fitting than the non-linear terms, justifying the moderately transformed slopes. We can further observe from the fitted curves that the outcome of PDT is less linear than that of PTT, with the latter tending to become more linear as the treatment duration increases. This is reflected by larger non-linear terms ( $I_{0}^{2}$ and *τ* ^2^) for PDT in comparison to PTT.

The error values for this model were -0.02 ± 12 and 0.01 ± 10 percent, but the model performed well, with R^2^ = 0.89 and 0.92 for PDT and PTT, respectively. The relatively low mean error can be attributed to the optimization criterion, which minimizes mean error over all fitted curves. However, due to the limitation on the number of regression terms, the fit was unable to capture all non-linearities, resulting in relatively high residuals for each training datapoint. The relatively high R^2^ value for the non-linear analytical fitting approach is expected, due to the monolithic nature of the data and the method of curve-fitting.

The results of the error analysis for training data predictions are summarized in **Table 1**. While the regression and interpolation methods appear to produce more accurate predictions than the analytical function method, the latter has the advantage of accessibility to clinicians and can also be further analyzed for sensitivity testing. However, where computational resources are unlimited, it seems that the interpolation method with low- pass filtering has the potential to achieve the highest accuracy.

**Table 1:** Training error analysis results, goodness of fit.

Treatment type		PDT		PTT
Prediction model	Residual error	R^2^	Residual error	R^2^
LOWESS Regression	4.8+11.0	0.89	3.8+10	0.90
Thin plate interpolation	0.0	1.00	0.0	1.00
Analytical model	0.0+0.1	0.89	0.0+0.1	0.92

### 3.4 Model verification and error analysis

**Figure 7:**
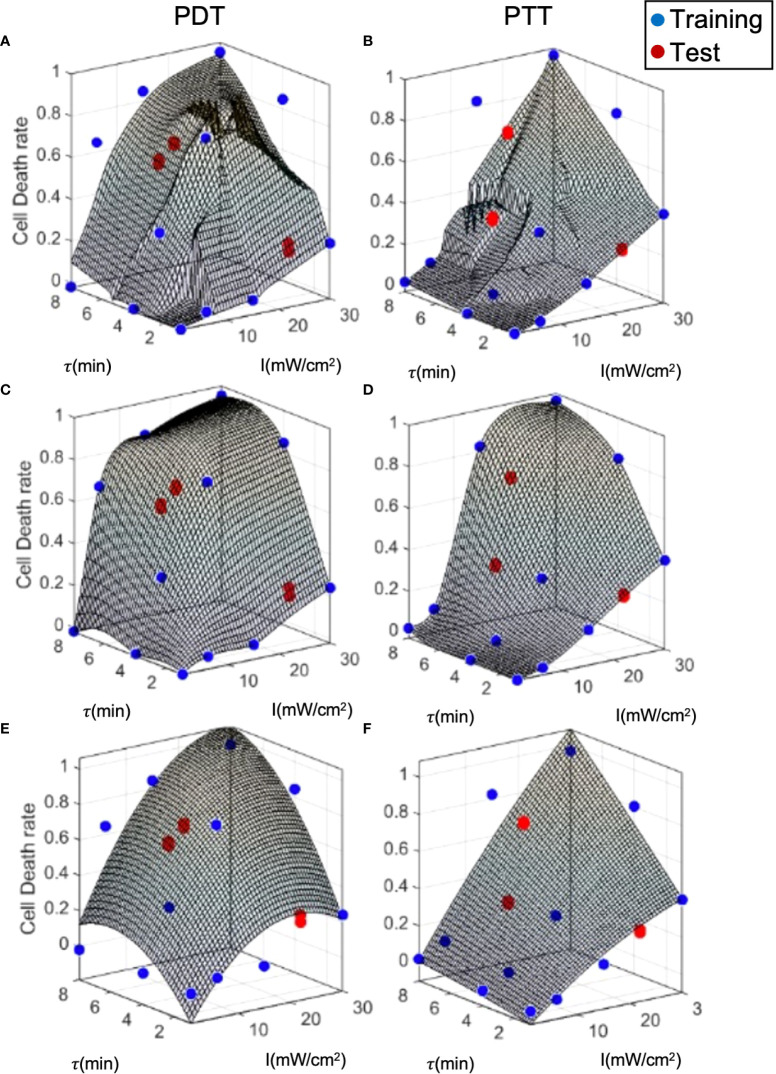
Model prediction error analysis for the three prediction methods. **(A, B)** LOWESS regression, **(C, D)** interpolation, **(E, F)** analytical function.

To examine model performance, we used a separate test dataset. The test datapoints are illustrated in **Figure 7**, where they can be compared to the training datapoints and the predicted curve generated by each of the three prediction models. The mean, standard deviation error, and R^2^ for each model with respect to the test data are summarized in **Table 2**. Overall, the error at test was significantly higher than the training error, as expected. In addition, outcome estimates were less accurate for PDT compared to PTT, due to the more complex shape of the PDT curve in relation to treatment duration. In the case of LOWESS regression, the error at test was twice as high as the training error, with similar standard deviation values. The mean error at test for the interpolation model was ≈20% and ≈10% for PDT and PTT, respectively. This was higher than the analytical function fitting model, which generated an error of ≈20% and ≈3% for the PDT and PTT, respectively. The regression model produced a lower mean error at test, but this was associated with a high standard deviation. The relatively high error values at test can be explained by a small degree of overfitting to the training datapoints, which constrained the curve-fitting and the model’s predictions. With the addition of more training data points, prediction error should also be minimized in the case of the interpolation model. The R^2^ values for the two treatments were highest in the case of the interpolation model, poorest in the case of the regression model, and mid-range in the case of the analytical function approximation. Similarly, considering the usability of the analytical curve, we can conclude that the analytical function offers reasonable performance with high usability. This set of results is expected on the basis of the theoretical properties of each of the three prediction models. The parameter tuning employed in the analytical function method results in a smoother curve due to the minimization of error over all data points, while the interpolation method generates the highest local errors as measured by R^2^, and the regression model produces the best local error results as a result of the regression cost function criterion. Higher- order analytical function fitting can provide even greater performance, with sufficient usability.

**Table 2:** Test error analysis results, goodness of fit.

Treatment type		PDT	PTT	
Prediction model	Residual error	R^2^	Residual error	R^2^
LOWESS Regression	11.9+8.2	0.82	-6.9+13.9	0.58
Thin plate interpolation	20.0+5.3	0.93	9.9+6.3	0.92
Analytical model	19.5#8.2	0.82	3.53+11.8	0.70

### 3.5 Approximation of cell state

To evaluate and calculate the probability of a given cell falling into each state after treatment, we present the cell death rate predictions of each model on two dimensions, as a function of laser radiation intensity and duration, in **Figure 7**. In a similar way to a three-dimensional representation, this illustrates the locality of the predictions of the regression model compared to the other two models. The analytical function model appears to be more similar to the regression model in the case of PTT, and more similar to the interpolation model in the case of PDT. Nevertheless, for PDT, the cell death region has a more rectangular shape.

**Figure 8:**
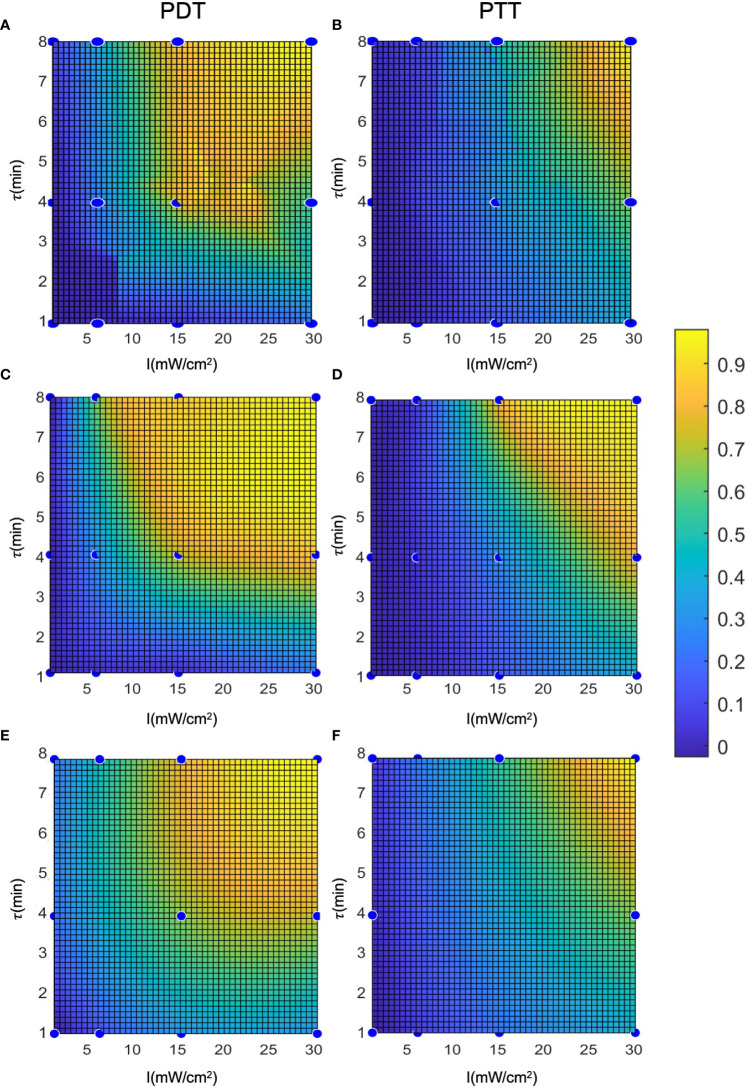
Representation of the cell death rate in two dimensions: **(A, B)** LOWESS regression, **(C, D)** interpolation, **(E, F)** analytical function.

Clustering can be applied to the outputs of the prediction models (**Figure 8**) to derive the probabilities of cells being unaffected, affected, or dead conditional on the treatment parameters. This clustering takes a different form to the clustering process described in (12), where the cell state probabilities *P_d_*, *P_a_*, and *P_na_* were determined in relation to the physiological properties of the cells after treatment. In this case, the clustering is based on the initial *P_d_*, with the prediction model outputs over the entire cell population and two repetitions of the experiment. The objective was to estimate the cell state probabilities as a function of the conditions applied in the laser radiation treatment.

**Figure 9:**
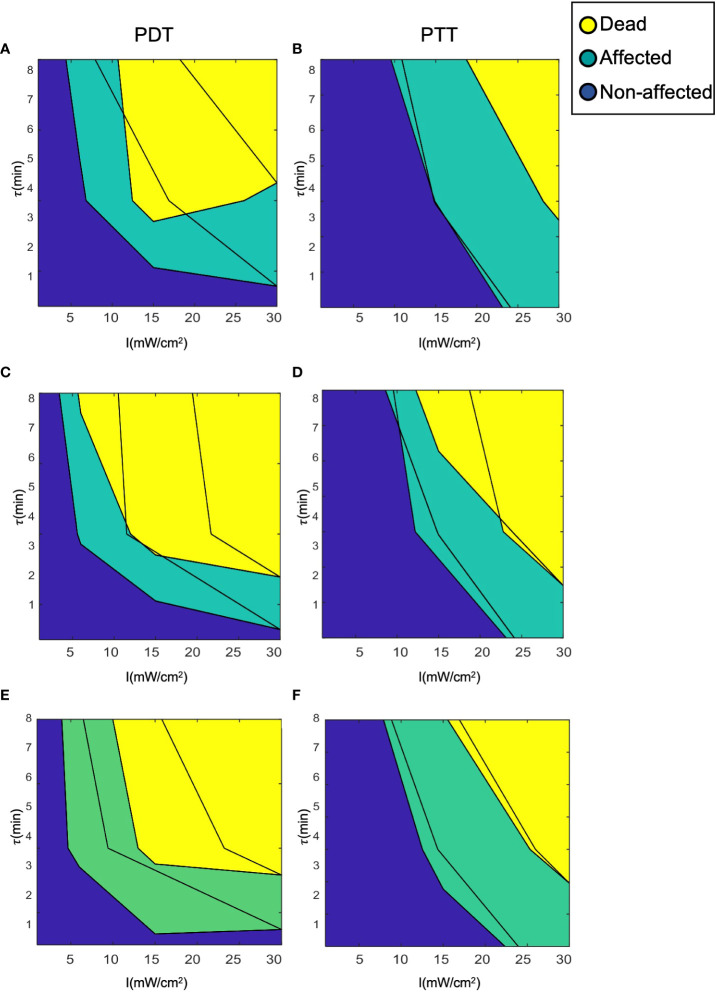
Clustering of cells into the three clinical classes (dead, affected, and unaffected). **(A, B)** LOWESS regression, **(C, D)** interpolation, **(E, F)** analytical function.

**Figure 9** shows an example of clustering of the cells with a cell death threshold of greater than 60% of the cell population (*D_TH_* = 0.6), an affected cell population between 30 and 60% (*H_TH_* = 0.3), and a proportion of live cells of less than 30%. The unaffected cell population (live cells) was pre-specified in such a manner as to ensure that the median population would be comparable to that of the control samples (≈ 3% death rate). The high degree of overlap between the clusters for the three prediction models appears to reduce prediction error compared to the continuous model. This can be explained by the filtering- like effect of the clustering process, which is based on cell population statistics and exclusion of artifacts (51). The clusters can be cross- validated by tracking cell survival rate or by observing the properties of the cells, as in Galluzzi etal. (53). This would determine the sensitivity and specificity of the threshold values. Cluster validation through additional experiments can also be used to measure prediction strength (54).

### 3.6 Gain from combined treatments

Combining multiple cancer treatments is essential in cancer therapy. The gain from doing so is equal to the relative gain for the application of two consecutive treatments compared with continuation of the first treatment. In this case, we examined identical laser radiation PDT and PTT treatments of 4- and 8 -minute durations for a fair comparison with the results of combined PDT and PTT treatment. Subsequently, we estimated a lower bound for the predicted gain (representing additive treatment gain), $B_{L}\left(D_{P_{n_{1}},P_{n_{2}}}^{n_{1}+n_{2},T}\right)$ , based on estimations of the results of PDT and PTT separately as in (18).

For the first dataset, in which treatment duration was 4 minutes, the cell death rates in the controls were $D_{\tau_{PDT}=4}^{PDT,24}=29.53\pm 7.32\%$ and $D_{\tau_{PTT}=4}^{PTT,24}=29.96\pm 1.98\%$ , and the rate for the combined treatment was $D_{\tau_{PDT}=2,\tau_{PTT}=2}^{PDT+PTT,24}=44.21\pm 2.57\%$ . For the second dataset, in which treatment duration was 8 minutes, the corresponding cell death rates were $D_{\tau_{PDT}=8}^{PDT,24}=66.33\pm 3.28\%$ and $D_{\tau_{PTT}=8}^{PTT,24}$ =81.16±4.75% for the controls, and $D_{\tau_{PDT}=4,\tau_{PTT}=4}^{PDT+PTT,24}=82.1\pm 6.13\%$ for the combined treatment.

For the first dataset, PDT and PTT were administered separately for a duration of 4 minutes in each case, and in the combined condition, each was administered for a duration of 2 minutes (25). For the second dataset, each treatment was administered separately for a duration of 8 minutes, and in the combined condition, each was administered for a duration of 4 minutes. In both data sets, the laser radiation intensity was set at 6 and 15 mW/cm^2^ for PDT and PTT, respectively. The lower bound estimated for the cell death rate is reported for the regression, interpolation, and analytical function models (LB1, LB2, LB3).

**Figure 10:**
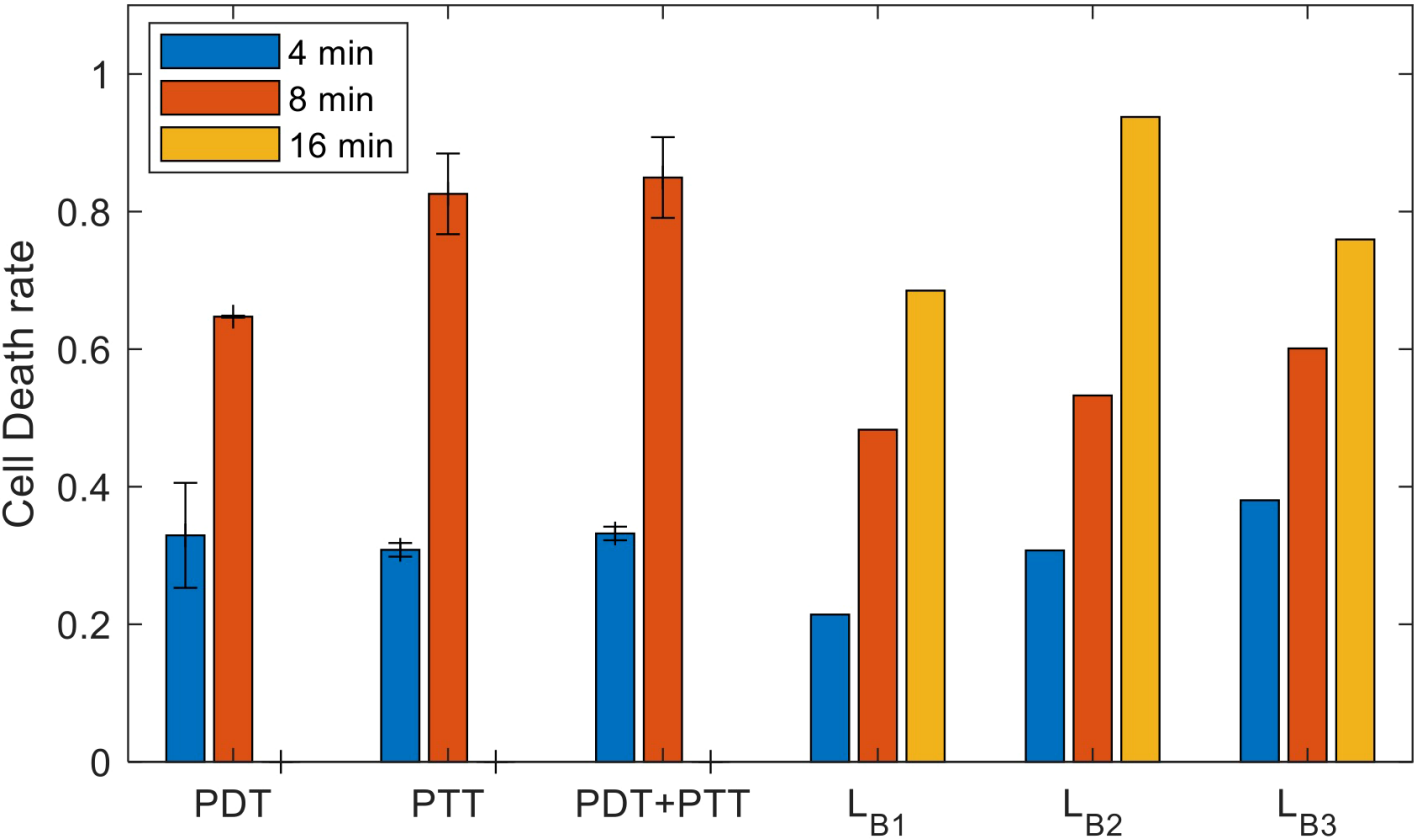
Experimental results for the sets of experiments with treatment durations of 4, 8, and 16 minutes (mean and inter-session standard deviation); estimated lower bounds for the outcome of combined treatment at durations of 4, 8, and 16 minutes.

The results of the experiment, in terms of mean and standard deviation of the cell death rate, along with the mean values of the estimated lower bounds for the combined treatment, are shown in **Figure 10**. As expected, we observed a significantly higher cell death rate following the more prolonged treatment. The increase in the efficiency of the treatment under this setup, at the predetermined range of intensities, is greater than the linear response that can be explained by the accumulation effect (27). The lower bounds calculated using each model presume an additive effect; these lower bounds are significantly lower than the experimental results for the lengthier 8- minute treatment in the case of all three treatments (PDT, PTT, and combined). This can be explained by inertial processes that cause cell death, which are not considered in calculating the lower bounds. Using the data for the combined treatment, or alternatively estimating the probability that a given cell will be affected, would be a possible way to achieve a closer estimate of the lower bound. The lower bounds calculated based on an additive effect (assuming there are no affected cells) are more accurate for the short- duration treatment (4 minutes), due to the relatively linearity of the curve in this region. At higher intensities, the bounds are less accurate due to non-linear accumulation of the treatment effect on the cells.

To determine the type of gain arising from the combined treatment (synergetic, additive, or antagonistic) and to quantify this gain, we substituted the results in (14). The results of doing so indicated gains of *S_G,_*_4_ (*PDT*) = 14.68±4.9 and *S_G_*,_4_ (*PTT*) = 47-23 = 14.25±2.1 for the combined treatment compared to PDT and PTT separately; these values represent synergetic gains of 49 and 47 percent, respectively. The results indicated that there was a high synergetic gain for shorter treatment durations. For the longer treatment duration of 8 minutes, the gains were *S_G_*_,8_ (*PDT*) = 15.77±4.2 and *S_G,_*_8_ (*PTT*)=0.94±5.4, values which represent a synergetic gain of 23 percent for PDT and 1 percent for PTT.

The results imply that there is a substantial synergetic effect for PDT, at both long and short treatment durations. In contrast, PTT treatment provides only an additive combined gain at long treatment durations. This indicates that, following 4 minutes of PTT treatment, there is no expected difference in outcome between switching to PDT and resuming PTT. This finding can be explained by a high death rate among damaged cells that do not undergo recovery under both the combined and the PTT treatments. The elevated efficiency of the PTT treatment when the NPs are heated to an elevated temperature is close to saturation, causing significant damage to the cell. Thus, damaged cells can be expected to die at similar rates under both treatments. Following this, the influence of the second treatment is restricted to unaffected cells, and is therefore additive.

## 4 Discussion and future work

In this article we provide a framework for a dual cancer treatment combining laser radiation with light- sensitive nanoparticles. After administering *in vitro* dual phototherapies (PDT and PTT) to SH-SY5Y cells, we modeled the efficiency and selectivity of both treatments. We used NPs that have been shown to be optimized for size and shape, and to be biocompatible. Subsequently, in order to maximize the treatment efficiency, we focused on optimization of the fundamental treatment parameters to identify the minimal laser radiation intensity and treatment duration that can be used to amplify the cancer cell death rate and the overall treatment efficiency.

We constructed several biomedical statistical models and defined three cell state probabilities: affected, unaffected, and dead. These probabilities were used to model the gain achieved by administering multiple consecutive treatments (combined treatment gain). We hypothesize that affected cells respond to subsequent treatment to a greater extent, consequently producing a synergetic effect. Following this analysis, we defined an optimization criterion based on the biomedical model.

We have demonstrated the feasibility of using a computational model and methods for modeling of combined laser radiation and NPs in the context of two new dual treatments (PDT and PTT) and their combination. Since there was no available dataset on which to train our models, we created a data set to solve this issue. Subsequently, to identify the optimal parameters for laser radiation, we constructed three prediction models that can provide predictions for continuous values on the basis of training data: a regression model, an interpolation model, and a model using fitting of an analytical function. We computed the performance of each model using error analysis on the test data. The preliminary results showed that the performance of all three models was sufficient, with death rate error values of 0.09, 0.15, and 0.12 for the regression, interpolation, and analytical function fitting approaches, respectively. However, due to its modest form, the analytical function model has a clinical advantage and can be further used for sensitivity analysis of the effect of the treatment parameters on performance.

In all, this framework constitutes a first step toward a toolkit for medical decision-making that can support the clinician in optimizing the use of separate or combined treatments involving a variety of cancer treatment methods, particularly PDT and PTT.

In the future, we plan to collect more *in vitro* data, add further treatment setup parameters (alongside radiation duration and intensity), enhance the classification accuracy of the model using deep learning networks, use enhanced *in vitro* 3D models to predict the effects of treatment under conditions more similar to an *in vivo* setting, and eventually apply our computational model to predict the efficiency of other cancer treatment combinations and examine its performance based on experiments involving clinical data.

## Data availability statement

The raw data supporting the conclusions of this article will be made available by the authors, without undue reservation.

## Author contributions

Conceptualization, EV, GB and OS; methodology, EV, GB and OS; software, EV and GB; validation, EV and GB; formal analysis, EV, and GB; investigation, data curation, EV, and GB; writing—original draft preparation, EV, GB, and OS; writing—review and editing, EV, GB, and OS; supervision, OS; project administration, OS; funding acquisition, OS. All authors have read and agreed to the published version of the manuscript.
